# Supplementary material for: A computational model of spatial politics: Hotelling-downs model as statistical physics
Source: PLoS One. 2026 Jun 23;21(6):e0352242. doi: 10.1371/journal.pone.0352242 (PMC13289859; doi:10.1371/journal.pone.0352242)
Supplement: Attachment — Submitted filename: ResponsetoReview.pdf [file pone.0352242.s001.pdf]

Response to referees' comments for manuscript

Dear Editor,

Thank you for the comments of the referees,

Major Changes

1. We have written a new code which seeks Nash Equilibria in the opinion space. This code provides a quasi-independent verification of the original.
2. We have fully rewritten the methods section to provide a coherent description of the two codes.
3. We extended the investigation of the two-dimensional case where, for single-peaked distribution on each issue (marginal distribution), the overall space is double-peaked.
4. we added a new figure to show the equilibrium positions.
5. We extended the discussion along the lines suggested by the referee

Detailed Response to Referee 1

Thank you for the opportunity to review this manuscript. This paper presents an interesting model of political party behavior that holds promise in analyzing several political phenomena in recent years, including: polarization, emergence of new political parties, and (in some cases) movement to the political center. While I am not an expert in political science, I agree with the authors that relatively simple models that demonstrate the ability to explain a variety of emergent phenomena can be quite useful theory-building tools, particularly when the mechanisms are difficult to explore using observed data and empirical methods.

I am generally enthusiastic about the potential contributions of the kind of model developed in this manuscript. However, I think there are a few aspects of the paper that currently obscure its contributions to political science and social systems modelling. Below, I detail some general issues that I think the authors could improve on, followed by a few specific requests for clarifications and minor suggestions. My sense is that these suggestions will be fairly feasible to address (or otherwise counter with compelling reasons for not including

in this manuscript), so I would welcome the opportunity to read a revised version of this manuscript.

We thank Prof Choquette-Levy for the positive response and the careful and detailed critique.

General Comments: *1. Incorporation of stochasticity in the model: While I generally agree with the claim that probabilistic models can provide valuable insights beyond what can be demonstrated with an analytical model (Lines 111-120 and Lines 312-315), I don't think the current manuscript clearly illustrates this. There is little discussion of the sources of stochasticity in this model, transient dynamics, uncertainty ranges, or other hallmarks of stochastic simulation models that can demonstrate added value beyond a cleaner analytical model. Throughout the manuscript, I would suggest the authors consider elaborating on a few of these concepts, notably:*

Incorporation of stochasticity. The description of probabilistic movement in Lines 121-124 is helpful, but this could be further elaborated. For example, are all positions in opinion space equally likely to be drawn in any given time step? Do parties move completely from V_1 to V_2 if the probability exceeds some random number? Or do they make a partial move to V_2 (which might be more realistic, and perhaps capture some internal attractors that wouldn't be uncovered with large movements)? Further, a sensitivity to the volatility parameter T would be illustrative. Are there non-linear relationships between T and the steady-state behavior that emerge?

We apologise for the lack of detail: it is well known that the Metropolis algorithm gives a distribution which is insensitive to the type of noise. However, the noise strongly affects the dynamics and convergence rates. We now say explicitly "parties trial new positions displaced from their current position by a vector drawn from a Gaussian distribution (variance 0.02 in the domain [-1,1]), and the change in vote share is calculated." This small variance follows Downs' approach in preventing parties making large "leaps" in the opinion space.

In detail, the steps are drawn from a multivariate normal and the positions are floats (the voters are on a grid but the parties are not). That normal distribution is centred at zero and the step is added to the current position, so in effect a normal distribution centred on the current position with variance 0.02 in domain [-1,1]. There is no bound on those steps, but if the new position is outside of the grid's boundaries it is automatically rejected.

- *Equilibrium vs. Steady-State results. The manuscript refers in several parts to find-*

ing "equilibria" of party behavior, but is it truly possible to prove these are equilibria in a probabilistic model? My understanding is that equilibria can only be proven in an analytical, differentiable model (though if I am incorrect, some clarification might be helpful in the Methods). This isn't to diminish the value of probabilistic models, but it seems like a more appropriate term for the results deriving from this model would be "steady-state" or "long-term outcome".

Thank you for bringing this up. "Equilibrium" is defined differently in different fields and it is good to have the chance to clarify it here. We specifically point out the difference between Nash equilibrium, static equilibrium (local minimum) and dynamic equilibrium (distribution). The referee is right that in an analytical, differentiable model a static equilibrium point is easily proven (e.g. the Hotelling two-party solution). In a stochastic system, the "dynamic equilibrium" refers to a probability distribution: an ensemble of possible states, each weighted by the probability of finding that state. Properties of such an "equilibrium" are found by integrating over those states, which we do here by stochastic sampling. One example of this would be a gas, where it is not necessary (or correct) to specify the position and velocity of every atom to prove that equilibrium exists.

We added a section discussing the static equilibrium in the Downs model.

Value-add of probabilistic models in this setting. The authors claim that probabilistic models can "find solutions which cannot be found analytically alone" (Lines 119-120), but I'm not sure that the current manuscript demonstrates any examples of this. If the model depends on two parameters and simulates the decisions of only 2-3 parties, would it not be feasible to conduct the same analysis analytically? Some clearer explanation of which results could only be found through a simulation model seems warranted.

This is related to the previous point: the stochastic sampling locates the equilibrium distribution which is typically centered around a given point. The referee is probably right that, in principle, there are solutions to the transcendental equations arising from equations 1-3. More accurate would be to say that we were unable to find analytic solutions and that there is no guarantee that such solutions exist, and for that reason we resorted to numerical methods.

Stochasticity in Results. Perhaps most importantly, if the current model does incorporate useful sources of stochasticity, I would encourage the authors to take better advantage of this in presenting their results. For example, only cumulative and steady-state results are dis-

played in the figures, but perhaps some discussion of transient dynamics would be instructive (even if the time steps don't correspond to "real world" time). Are there certain types of pathways through opinion space that parties take under different parameter combinations, and can these be illustrated (e.g., through gradient diagrams)? What might be the implications of those pathways? For example, if parties converge in opinion space before diverging, perhaps this might signal a limited window for a grand coalition that mitigates polarization. Furthermore, if the model is run through several stochastic simulations, some illustration of uncertainty ranges and their interpretation could be useful. It wasn't clear to me if the histograms on Figs. 2 and 3 demonstrate results over multiple simulations or over just one simulation, and it seems to me that Figs. 4-6 all should have some uncertainty associated with the illustrated relationships, assuming there is some randomness in the model.

2. Elaboration of model mechanisms: One strength of this manuscript is its clarity and conciseness regarding the mechanisms tested in this version of the Hotelling-Downs model. I found the discussion of turnout to be particularly strong: while not immediately intuitive, the non-linear relationship between turnout and polarization seems to be a valuable insight from this kind of model.

I found the discussion of "activists" to be less compelling, mostly because this seems to be somewhat of a tautology. My understanding of the way this was incorporated in the model is that if the activism parameter is increased, voters on the extremes are accorded greater weight, and therefore parties become more polarized because they are pulled to the extremes. This seems fairly self-evident and a bit of a "cheat code" to increase polarization in the model.

We thank the reviewer for the positive comments about turnout and its not completely intuitive effects. Regarding the "activists", it might seem tautological that increased activism causes polarisation, but we note that this does not occur in the simple 1D Hotelling model unless the activism parameter is strong. In fact, it is the interplay between turnout and activism which first splits the party positions through turnout, only then exacerbates it through activism.

Furthermore, we argue that the effect is real, and should be included, even if the outcome is rather obvious. Without it, we risk that polarization is attributed to a different, perhaps less important, cause.

However, the referee inspired us to create a model in which activism is different from

extremism. We now consider an alternative activist effect where the party position is attracted towards the average position of its voters (i.e. all supporters exert a pull on the party position). In this model there is no draw towards extreme positions, nevertheless, in almost all cases, the centroid of the voter polyhedron is more extreme than the optimal position, so this type of non-extreme activism also leads to more polarization.

I wonder if the model could be amended to demonstrate how this behavior emerges from more fundamental principles (that perhaps correspond more closely to real-world phenomena). For example, could there be an interesting dynamical component whereby voters that feel "closer" to a party in political space over a certain number of time steps steadily grow in weight (i.e., they are seen as the core of the party), or conversely, voters that feel most distant from any party grow in disaffection and weight over time (representing the threat of a new political movement)? This might still allow for activism to emerge, but from more fundamental principles whose outcomes aren't immediately intuitive. Further, it could be another example of how a stochastic/probabilistic model can offer insights beyond that of an analytical/deterministic model.

We have started thinking about how to do this, but it opens many possibilities and arbitrary choices. So, we feel it is beyond the scope of the paper, but we have developed the open-source code in a flexible way so that interested readers can easily extend it.

Finally, I was left wondering how the actions of one party affect the decisions taken by another party, if at all. Are there any direct or indirect game theoretic interactions in the way the model is set up? For example, if one party decides to move to the center and changes the boundaries of the Voronoi polyhedral, does this affect the optimal behavior of other parties (even assuming the same voter distribution)? And if so, does this represent another important source of stochasticity between different simulations?

There is an indirect interaction between the parties as the voronoi boundaries shift. Exactly how this plays out, i.e. whether the party moves closer to a rival to recapture those voters, or further away to look elsewhere for support, depends on the voter distribution.

3. General exposition and motivation of this model: The Introduction of the manuscript connects this model to existing canonical models (Hotelling's Law, Duverger's Law), but I think the applicability of the model presented here could be better motivated with a more robust introduction. First, while Hotelling's Law is clearly explained, Duverger's Law is only briefly mentioned and appears more relevant for the current work. Some more explanation

of this law and how the current manuscript builds on its theoretical groundwork would be helpful.

We discussed Duverger's Law - that ”first-past-the-post” voting systems tend to favour a two-party political system - in more detail. We do not directly consider effects of voting system here, but the number of parties is significant, and Duverger's Law provides a rationale for studying the two-party case. Self-evidently, ”First-past-the-post” underrepresents minority parties. Without turnout/activist effect we have - perhaps surprisingly - a tendency for two party systems to both move toward the centre, such that whichever is elected will closely represent average voters preference. Multiparty systems cover the opinion space more widely, meaning that individual voters will typically find a party that better represents their viewpoint. However, assuming the government is formed by parties with similar ethos - in the same region of phase space - the actual government will be less representative.

Second, while the authors attempt to make this model generalizable to multiple types of democratic systems (a laudable goal), the only concrete mentions of polarization are from U.S. case studies. While the global relationship between turnout and polarization (Fig. 7 and Lines 361-372) is a helpful start, I think the authors could better illustrate the generalizability of such a model by considering how well it might replicate other case studies of polarization – for example, the rightward shift of center-right parties in France and India, and/or increasing polarization of political parties in Chile and Colombia. Admittedly, I'm not sure to what extent data on turnout or other potential model parameters is available in these settings, but some discussion about whether there is any evidence that these mechanisms have indeed contributed to polarization in other settings can help improve the external validity of the model.

We are nervous about getting into discussion of cases which may expose political opinions. We note that in a previous work (for a different journal) the editor did not accept anything which might be viewed as expressing a political opinion. We have some sympathy for that viewpoint. It is also perfectly possible that the referee may disagree with our categorisation of specific parties. The one linking feature is the effect of new parties, which we discuss in a little more detail.

The drift to the right in France is well established (we now cite Ivaldi on this). However, evidence for issue polarization is weaker. e.g. in the extensive IPSOS survey *https://www.sciencespo.fr/cevipof/sites/sciencespo.fr.cevipof/files/Ipsos_ENEF_Vague7_Aout2024vlight.pdf*

many subjects are reviewed on a four-point scale: (Tout à fait favorable; Plutôt favorable; Plutôt pas favorable; Pas du tout favorable). In every case, the two most popular choices are adjacent (i.e unimodal). This suggests that there is no strong issue polarisation and the rightward drift may represent a rightward drift in the opinion space, which all parties would track. Our model of polarization in 2D due to correlations seems relevant here. As the model suggests, the far right "Rassemblement national" has obtained increasing success while moving towards the centre, while the centre right parties compete by moving right. The stablity of a far right party is supported in our model by reducing turnout, and our model suggest it facilitates the emergence of a far left equivalent. The system of top-2 runoffs means Duverger's law can be applied in France, leading to a bipolar competition (two coalitions) rather than a strict two-party system. Elsewhere, to some extent in France but particularly in India, it could be argued that the "rightward" drift is really an opening up of nationalism as a new axis in the opinion space. Certainly here in Scotland we have a strong Nationalist movement (SNP) which is leftist on most issues. Even the far-right "Reform" party are budgeting for increased state funding of our nationalised health system (NHS), widely regarded as the crowning achievement of UK progressive politics in the last century.

Chile and Colombia are different again, likely shaped by the acceptibility of armed "parties" - the rightist Pinochet in Chile and leftist FARC in Columbia.

What we can say from the model is the existence of extreme parties tends to cause polarisation in the centre, and increasing the dimensionality (i.e. number of independent issues affecting voter choice) can lead to party polarisation even if there is a consensus of all issues.

Specific Comments: Thank you for these. In places the replies are short, to avoid repetition of responses above. 1. Lines 28-29: Is there evidence that both major U.S. parties have become equally polarized, or is this asymmetric (i.e., one party shifts more to the extremes than the latter)?

According to a very recent analysis of American National Election Studies data (1988-2024 - i.e. prior to the current Trump administration) by Young et al

https://royalsocietypublishing.org/rsos/article/13/2/251428/479919/A-new-measure-of-issue-polarization-using-k-means. As one might expect, progressives have shifted their positions more than conservatives: in the US context Democratic supporters have moved

left while the Republican supporters have maintained their policy position. We now reference this paper which appeared after our original submission. This could be interpreted as Democrats being "more polarised" realtive to 1980's opinions. In our model, it would appear as a leftwards shift of the centre of the Overton window leaving the parties equally polarised..

2. Lines 63-65: I'd suggest that this might be a good place to be more specific about the contributions of the model. Stating that the model can accommodate multiple parties and multiple dimensions is good, but what are the main insights this generates (that wouldn't be garnered in a more traditional one-dimensional model)?

We thank the referee for this question, which we now consider more carefully. An important insight is that the 2D opinion space can be polarised (two peaked) even when each axis shows a consensus (single-peaked marginal distribution). As discussed, this polarisation comes from "party sorting".

3. Lines 95-98: The description of this assumption is helpful – but can you test other voter distributions with this model? This seems to be implied in the Results section (Lines 207-210), but some structured exploration of party behavior under different voter distributions could be a valuable contribution. Even if you impose a static distribution, it should be possible (and interesting) to compare how the mechanisms tested here affect polarization under different voter distribution skews.

This extremely insightful comment has inspired us to analyse the two-peaked distributions.

4. Lines 105-110: I agree this assumption is reasonable, and allowing voters to be maximally polarized in multiple dimensions seems like a valuable contribution of this study (perhaps worth emphasizing earlier on and/or in the Discussion).

We covered this and added a sentence explaining why the axes are meaningful in the context of acceptible viewpoints (Overton window).

5. Lines 114-116: This may be at the crux of my question regarding whether "equilibria" can be found in this type of probabilistic model. Some more background here on Metropolis Monte Carlo could be useful, including the core assumptions. If one simulates over a finite number of time steps, is there really assurance that this method replicates the expected value of a distribution?

There is no general proof that Metropolis Monte Carlo will replicate the distribution

in a given time. However, it is the standard method using in statistical physics and over 50,000 citations to the original paper stand testimony to its effectiveness. In general, it will converge slowly for very high dimensional systems with many, distinct maxima, but here we can easily visualise the trajectory in 2D and readily verify that the regions of high vote-count are well sampled. Repeated runs from different starting conditions find similar distributions. As a further check, we implemented a deterministic "equilibrium search" algorithm which finds as stable points the same regions around which the Monte Carlo moves.

6. Lines 173-177: As stated above, I'm not sure that the way activism is currently operationalized in the model is especially useful. But, if it is kept in the model, I think it would be important here to give justification for why activists should be accorded more weight. While this may seem sensible in certain salient examples of polarization, I think we could equally find examples throughout recent history of parties marginalizing factions they believe are too extreme. Overall though, I'd encourage the authors to consider some other mechanism that allows polarization to emerge from first principles.

We appreciate that giving extra weight to voters with extreme positions looks like putting the answer in. So we implemented a second approach to "activists" which gives all party voters equal weight. Again, this increases polarisation because the mean voter is typically further from the centre. Regarding "marginalizing factions they believe are too extreme": this is precisely what the Downs model assumes, the party moves i response to where all the voters are, not where its members are.

7. Lines 188-191: This seems like a reasonable approach, but it would be good to provide evidence of this, for example via a time-series plot that displays an example of party behavior dynamics (perhaps as an Appendix figure).

We added some time series plots. A caveat here is that most of the motion comes from moving away from the randomly chosen initial positions.

8. Lines 213-214: This is an interesting insight from the turnout experiments. Again, I wonder if a more systematic exploration of the effect of turnout under different voter distributions could help illustrate the nuanced conditions under which depressed turnout leads to more polarization (e.g., is there a threshold of voter distribution skew at which this no longer holds?).

We implemented the two-peaked model to investigate this, now described in detail throughout the text.

9. Lines 354-356: This is an interesting insight, but I think worth qualifying that this holds if barriers to vote are uniformly experienced across the political spectrum. If barriers to vote are instead biased towards one part of the spectrum (as seems to happen in real life), I'm not sure this conclusion holds.

We understand the point, and we agree with it. But this isn't what we meant! "Barriers to vote" was a bad choice of words implying the elections are not free. This would be extremely hard to model because it would require specifying exactly how barriers are non-uniform. What we really meant was that people weren't required to vote. We removed the word.

10. Lines 372-374: This is one helpful piece of evidence but I'm not sure it establishes a clear direction of causality. It could be equally plausible that voter turnout decreases because of increasing party polarization (unless Van-der-Veen establishes some causal direction, which would be worth mentioning here).

van der Veen suggests a causal relation, but it is not proven. To avoid falling foul of the correlation/causation fallacy, we replaced "leads to" with "is correlated with". We note that this study had the possibility to disprove our model is the correlation was in the opposite direction.
