# Supplementary material for: A computational model of spatial politics: Hotelling-downs model as statistical physics
Source: PLoS One. 2026 Jun 23;21(6):e0352242. doi: 10.1371/journal.pone.0352242 (PMC13289859; doi:10.1371/journal.pone.0352242)
Supplement: Attachment — Submitted filename: OpinionSpaceResponse2.pdf [file pone.0352242.s002.pdf]

Response to referees' comments for manuscript

Dear Editor,

We thank the reviewers for their positive responses and have implemented the minor suggestions from referee 1 as follows.

1. L33-35: This is a helpful description of different concepts of polarization in the Downs framework, and it would be useful to explicitly state which ones you are engaging with here (I think just the latter?).

Agreed. It is the latter, although later we do also touch on differences between more or less steep single-peaked distributions.

2. L62-67: Similarly, this is a helpful description of different types of equilibria, and it would be good to state which one(s) you use in this analysis.

Agreed and added.

3. L138-141: I found this description to be especially helpful in understanding the connection between turnout and activism in the model. This level of detail rightly belongs in the "Model" section, but I wonder if this conceptual link could be made earlier in the Introduction. That is, could you more clearly motivate why it's important to analyze turnout and activism jointly (e.g., pointing to the possibility that the effects of imperfect turnout on polarization are likely to depend on whether voters are uniformly weighted)? I think that might help readers more easily track the contributions of this multi-factor approach.

A sentence has been added to the introduction.

4. L58-173: This section is an interesting addition and makes sense to me statistically, but I was unclear about what "correlation" means in a political context here. Does this refer to correlation on a [-1,1] spectrum in voter perceptions on Issue X and Y? In other words, in a high-correlation condition, if voters tend to be more "left" on issue X, would they also be more "left" on issue Y? Perhaps an example here might help.

The referee understands it perfectly - statistical correlation in the 2D space. One might consider the Pearson correlation coefficient, but one can construct distribrution like Fig 3 with 3 or 4 peaks for which that formal measure is zero.

We give the following example: By extending the opinion space to two dimensions, we can investigate the effects on voting if two different issues are important, e.g. Economic and

Cultural on axes from left to right-wing and liberal/multicultural to traditional/nationalist. Each party must take a position of both issues, for example a left wing, nationalist party. The distribution of voters may then be *correlated*: e.g. there may be more left-wing nationalists than right-wing ones. We investigate such a correlated situation where on both issues there is a consensus (most voters in the centre), but the two dimensional space may have two peaks and show that, with only two parties, the Hotelling-consensus emerges, but with more parties they move to the peaks of the voter distribution.

5. Table 1 caption: This last sentence ("The 2D data is ... tending towards 0.765") didn't make sense to me. Why would a random placement necessarily tend to 0.765?

Thank you for pointing that out - is indeed far from obvious. It involves the integral for the mean distance of a randomly chosen point from the centre of the square, which is $\frac{1}{3}(\sqrt{2} + \ln(1 + \sqrt{2}) = 0.765$. We now added the equation.

6. Fig. 2: This is an intriguing comparison of equilibria positions for different numbers of parties. I think the example trajectories on the left-hand panel are helpful, but (at risk of contradicting myself from the initial review) they might be overkill for the right-hand panel with considerably more parties. Additionally, it might be helpful to include arrows showing the direction of the movement (I assume generally from the outside towards the centre of the circle). Finally, the caption mentions a legend for final vote share, but I think this has been omitted, at least from the image files in this submission.

We thank the referee for spotting the error that the vote shares are not given: they vary significantly from run to run so specific numbers are not informative nor reproducible; for each case, they can be inferred from the shaded area. We deleted the clause. We accept that the second figure with 20 parties is overkill for practical purposes, but we prefer to retain it to display a limiting case of the model. We explored using arrows, but we found that the "tadpole" plots with the head showing the final position are clearer, but to address the referees concern we have the explanatory text "Lines show typical trajectories from random starting locations, circles show final positions,"

*7. L 269-271: This is an interesting finding, and I wonder if there are also non-linearities in the turnout*activism interaction depending on the strength of the turnout factor. In other words, does activism change the critical value of for which polarization starts to decrease again? This might be grist for future work or reflections in the last part of the Discussion. But I thought it would be helpful to bring up here, as it might explain within-case study*

patterns whereby polarization increases even as voter turnout steadily decreases.

This is an interesting question but it requires an additional parameter which essentially put the answer in. The model is flexible enough to allow for this, but we feel it should be grounded in some fieldwork so we leave it for future work.

8. L 499-503: Slight quibble here. I understand the point that more parties may lead each individual party to be more extreme than a two-party system. But if parties on different ends of an issue are in a coalition government (arguably more likely in a system with several parties), I don't think this necessarily makes the government and government policy less representative of the overall median voter.

Interesting point on which we now comment. This argument is based around the premise "parties on different ends of an issue are in a coalition government". This may apply to some issues, but in general coalitions are formed between parties with similar ideologies.
