# Supplementary material for: A computational model of spatial politics: Hotelling-downs model as statistical physics
Source: PLoS One. 2026 Jun 23;21(6):e0352242. doi: 10.1371/journal.pone.0352242 (PMC13289859; doi:10.1371/journal.pone.0352242)
Supplement: Peer review history

# Peer review: Decision Letter 0

**DOI:** 10.1371/journal.pone.0352242.r001

**Authors:** Asim Zia

10 Feb 2026

PONE-D-25-53063

A computational model of spatial politics: Political models as statistical physics

PLOS One

Dear Dr. Ackland,

Thank you for submitting your manuscript to PLOS ONE. After careful consideration, we feel that it has merit but does not fully meet PLOS ONE’s publication criteria as it currently stands. Therefore, we invite you to submit a revised version of the manuscript that addresses the points raised during the review process.

Please submit your revised manuscript by Mar 27 2026 11:59PM. If you will need more time than this to complete your revisions, please reply to this message or contact the journal office at plosone@plos.org. When you're ready to submit your revision, log on to https://www.editorialmanager.com/pone/ and select the 'Submissions Needing Revision' folder to locate your manuscript file.

Please include the following items when submitting your revised manuscript:

- A letter that responds to each point raised by the academic editor and reviewer(s). You should upload this letter as a separate file labeled 'Response to Reviewers'.
- A marked-up copy of your manuscript that highlights changes made to the original version. You should upload this as a separate file labeled 'Revised Manuscript with Track Changes'.
- An unmarked version of your revised paper without tracked changes. You should upload this as a separate file labeled 'Manuscript'.

If you would like to make changes to your financial disclosure, please include your updated statement in your cover letter. Guidelines for resubmitting your figure files are available below the reviewer comments at the end of this letter.

If applicable, we recommend that you deposit your laboratory protocols in protocols.io to enhance the reproducibility of your results. Protocols.io assigns your protocol its own identifier (DOI) so that it can be cited independently in the future. For instructions see: https://journals.plos.org/plosone/s/submission-guidelines#loc-laboratory-protocols. Additionally, PLOS ONE offers an option for publishing peer-reviewed Lab Protocol articles, which describe protocols hosted on protocols.io. Read more information on sharing protocols at https://plos.org/protocols?utm_medium=editorial-email&utm_source=authorletters&utm_campaign=protocols.

We look forward to receiving your revised manuscript.

Kind regards,

Asim Zia, Ph.D.

Academic Editor

PLOS One

Journal Requirements:

When submitting your revision, we need you to address these additional requirements.

1. Please ensure that your manuscript meets PLOS ONE's style requirements, including those for file naming. The PLOS ONE style templates can be found at

https://journals.plos.org/plosone/s/file?id=wjVg/PLOSOne_formatting_sample_main_body.pdf and

https://journals.plos.org/plosone/s/file?id=ba62/PLOSOne_formatting_sample_title_authors_affiliations.pdf

2. If the reviewer comments include a recommendation to cite specific previously published works, please review and evaluate these publications to determine whether they are relevant and should be cited. There is no requirement to cite these works unless the editor has indicated otherwise.

Additional Editor Comments:

This manuscript has a lot of potential; however, Reviewer 1 has provided comprehensive set of recommendations that require to be addressed in the revised manuscript. In addition to the recommendations provided by Reviewer 1, an application of the proposed model to reproduce a country-specific polarization & voting data (e.g., USA, France and/or India etc.) would enhance the potential impact of this article.

[Note: HTML markup is below. Please do not edit.]

Reviewers' comments:

Reviewer's Responses to Questions

**Comments to the Author**

1. Is the manuscript technically sound, and do the data support the conclusions?

The manuscript must describe a technically sound piece of scientific research with data that supports the conclusions. Experiments must have been conducted rigorously, with appropriate controls, replication, and sample sizes. The conclusions must be drawn appropriately based on the data presented.

Reviewer #1: Yes

**********

2. Has the statistical analysis been performed appropriately and rigorously?

Reviewer #1: Yes

**********

3. Have the authors made all data underlying the findings in their manuscript fully available?

The PLOS Data policy requires authors to make all data underlying the findings described in their manuscript fully available without restriction, with rare exception (please refer to the Data Availability Statement in the manuscript PDF file). The data should be provided as part of the manuscript or its supporting information, or deposited to a public repository. For example, in addition to summary statistics, the data points behind means, medians and variance measures should be available. If there are restrictions on publicly sharing data—e.g. participant privacy or use of data from a third party—those must be specified.

Reviewer #1: Yes

**********

4. Is the manuscript presented in an intelligible fashion and written in standard English?

PLOS ONE does not copyedit accepted manuscripts, so the language in submitted articles must be clear, correct, and unambiguous. Any typographical or grammatical errors should be corrected at revision, so please note any specific errors here.

Reviewer #1: Yes

**********

5. Review Comments to the Author

Please use the space provided to explain your answers to the questions above. You may also include additional comments for the author, including concerns about dual publication, research ethics, or publication ethics. (Please upload your review as an attachment if it exceeds 20,000 characters)

Reviewer #1: Thank you for the opportunity to review this manuscript. This paper presents an interesting model of political party behavior that holds promise in analyzing several political phenomena in recent years, including: polarization, emergence of new political parties, and (in some cases) movement to the political center. While I am not an expert in political science, I agree with the authors that relatively simple models that demonstrate the ability to explain a variety of emergent phenomena can be quite useful theory-building tools, particularly when the mechanisms are difficult to explore using observed data and empirical methods.

I am generally enthusiastic about the potential contributions of the kind of model developed in this manuscript. However, I think there are a few aspects of the paper that currently obscure its contributions to political science and social systems modelling. Below, I detail some general issues that I think the authors could improve on, followed by a few specific requests for clarifications and minor suggestions. My sense is that these suggestions will be fairly feasible to address (or otherwise counter with compelling reasons for not including in this manuscript), so I would welcome the opportunity to read a revised version of this manuscript.

General Comments:

1. Incorporation of stochasticity in the model: While I generally agree with the claim that probabilistic models can provide valuable insights beyond what can be demonstrated with an analytical model (Lines 111-120 and Lines 312-315), I don’t think the current manuscript clearly illustrates this. There is little discussion of the sources of stochasticity in this model, transient dynamics, uncertainty ranges, or other hallmarks of stochastic simulation models that can demonstrate added value beyond a “cleaner” analytical model. Throughout the manuscript, I would suggest the authors consider elaborating on a few of these concepts, notably:

• Incorporation of stochasticity. The description of probabilistic movement in Lines 121-124 is helpful, but this could be further elaborated. For example, are all positions in opinion space equally likely to be drawn in any given time step? Do parties move completely from V_1 to V_2 if the probability exceeds some random number? Or do they make a partial move to V_2 (which might be more realistic, and perhaps capture some internal attractors that wouldn’t be uncovered with large movements)? Further, a sensitivity to the volatility parameter T would be illustrative. Are there non-linear relationships between T and the steady-state behavior that emerge?

• Equilibrium vs. Steady-State results. The manuscript refers in several parts to finding “equilibria” of party behavior, but is it truly possible to prove these are equilibria in a probabilistic model? My understanding is that equilibria can only be proven in an analytical, differentiable model (though if I am incorrect, some clarification might be helpful in the Methods). This isn’t to diminish the value of probabilistic models, but it seems like a more appropriate term for the results deriving from this model would be “steady-state” or “long-term outcome”.

• Value-add of probabilistic models in this setting. The authors claim that probabilistic models can “find solutions which cannot be found analytically alone” (Lines 119-120), but I’m not sure that the current manuscript demonstrates any examples of this. If the model depends on two parameters and simulates the decisions of only 2-3 parties, would it not be feasible to conduct the same analysis analytically? Some clearer explanation of which results could only be found through a simulation model seems warranted.

• Stochasticity in Results. Perhaps most importantly, if the current model does incorporate useful sources of stochasticity, I would encourage the authors to take better advantage of this in presenting their results. For example, only cumulative and steady-state results are displayed in the figures, but perhaps some discussion of transient dynamics would be instructive (even if the time steps don’t correspond to “real world” time). Are there certain types of pathways through opinion space that parties take under different parameter combinations, and can these be illustrated (e.g., through gradient diagrams)? What might be the implications of those pathways? For example, if parties converge in opinion space before diverging, perhaps this might signal a limited window for a grand coalition that mitigates polarization. Furthermore, if the model is run through several stochastic simulations, some illustration of uncertainty ranges and their interpretation could be useful. It wasn’t clear to me if the histograms on Figs. 2 and 3 demonstrate results over multiple simulations or over just one simulation, and it seems to me that Figs. 4-6 all should have some uncertainty associated with the illustrated relationships, assuming there is some randomness in the model.

2. Elaboration of model mechanisms: One strength of this manuscript is its clarity and conciseness regarding the mechanisms tested in this version of the Hotelling-Downs model. I found the discussion of turnout to be particularly strong: while not immediately intuitive, the non-linear relationship between turnout and polarization seems to be a valuable insight from this kind of model.

I found the discussion of “activists” to be less compelling, mostly because this seems to be somewhat of a tautology. My understanding of the way this was incorporated in the model is that if the activism parameter is increased, voters on the extremes are accorded greater weight, and therefore parties become more polarized because they are pulled to the extremes. This seems fairly self-evident and a bit of a “cheat code” to increase polarization in the model.

I wonder if the model could be amended to demonstrate how this behavior emerges from more fundamental principles (that perhaps correspond more closely to real-world phenomena). For example, could there be an interesting dynamical component whereby voters that feel “closer” to a party in political space over a certain number of time steps steadily grow in weight (i.e., they are seen as the core of the party), or conversely, voters that feel most distant from any party grow in disaffection and weight over time (representing the threat of a new political movement)? This might still allow for activism to emerge, but from more fundamental principles whose outcomes aren’t immediately intuitive. Further, it could be another example of how a stochastic/probabilistic model can offer insights beyond that of an analytical/deterministic model.

Finally, I was left wondering how the actions of one party affect the decisions taken by another party, if at all. Are there any direct or indirect game theoretic interactions in the way the model is set up? For example, if one party decides to move to the center and changes the boundaries of the Voronoi polyhedral, does this affect the optimal behavior of other parties (even assuming the same voter distribution)? And if so, does this represent another important source of stochasticity between different simulations?

3. General exposition and motivation of this model: The Introduction of the manuscript connects this model to existing canonical models (Hotelling’s Law, Duverger’s Law), but I think the applicability of the model presented here could be better motivated with a more robust introduction. First, while Hotelling’s Law is clearly explained, Duverger’s Law is only briefly mentioned and appears more relevant for the current work. Some more explanation of this law and how the current manuscript builds on its theoretical groundwork would be helpful. Second, while the authors attempt to make this model generalizable to multiple types of democratic systems (a laudable goal), the only concrete mentions of polarization are from U.S. case studies. While the global relationship between turnout and polarization (Fig. 7 and Lines 361-372) is a helpful start, I think the authors could better illustrate the generalizability of such a model by considering how well it might replicate other case studies of polarization – for example, the rightward shift of center-right parties in France and India, and/or increasing polarization of political parties in Chile and Colombia. Admittedly, I’m not sure to what extent data on turnout or other potential model parameters is available in these settings, but some discussion about whether there is any evidence that these mechanisms have indeed contributed to polarization in other settings can help improve the external validity of the model.

Specific Comments:

1. Lines 28-29: Is there evidence that both major U.S. parties have become equally polarized, or is this asymmetric (i.e., one party shifts more to the extremes than the latter)?

2. Lines 63-65: I’d suggest that this might be a good place to be more specific about the contributions of the model. Stating that the model can accommodate multiple parties and multiple dimensions is good, but what are the main insights this generates (that wouldn’t be garnered in a more traditional one-dimensional model)?

3. Lines 95-98: The description of this assumption is helpful – but can you test other voter distributions with this model? This seems to be implied in the Results section (Lines 207-210), but some structured exploration of party behavior under different voter distributions could be a valuable contribution. Even if you impose a static distribution, it should be possible (and interesting) to compare how the mechanisms tested here affect polarization under different voter distribution skews.

4. Lines 105-110: I agree this assumption is reasonable, and allowing voters to be maximally polarized in multiple dimensions seems like a valuable contribution of this study (perhaps worth emphasizing earlier on and/or in the Discussion).

5. Lines 114-116: This may be at the crux of my question regarding whether “equilibria” can be found in this type of probabilistic model. Some more background here on Metropolis Monte Carlo could be useful, including the core assumptions. If one simulates over a finite number of time steps, is there really assurance that this method replicates the expected value of a distribution?

6. Lines 173-177: As stated above, I’m not sure that the way activism is currently operationalized in the model is especially useful. But, if it is kept in the model, I think it would be important here to give justification for why activists should be accorded more weight. While this may seem sensible in certain salient examples of polarization, I think we could equally find examples throughout recent history of parties marginalizing factions they believe are too extreme. Overall though, I’d encourage the authors to consider some other mechanism that allows polarization to emerge from first principles.

7. Lines 188-191: This seems like a reasonable approach, but it would be good to provide evidence of this, for example via a time-series plot that displays an example of party behavior dynamics (perhaps as an Appendix figure).

8. Lines 213-214: This is an interesting insight from the turnout experiments. Again, I wonder if a more systematic exploration of the effect of turnout under different voter distributions could help illustrate the nuanced conditions under which depressed turnout leads to more polarization (e.g., is there a threshold of voter distribution skew at which this no longer holds?).

9. Lines 354-356: This is an interesting insight, but I think worth qualifying that this holds if barriers to vote are uniformly experienced across the political spectrum. If barriers to vote are instead biased towards one part of the spectrum (as seems to happen in real life), I’m not sure this conclusion holds.

10. Lines 372-374: This is one helpful piece of evidence but I’m not sure it establishes a clear direction of causality. It could be equally plausible that voter turnout decreases because of increasing party polarization (unless Van-der-Veen establishes some causal direction, which would be worth mentioning here).

**********

6. PLOS authors have the option to publish the peer review history of their article (what does this mean?). If published, this will include your full peer review and any attached files.

If you choose “no”, your identity will remain anonymous but your review may still be made public.

**Do you want your identity to be public for this peer review?**    For information about this choice, including consent withdrawal, please see our Privacy Policy.

Reviewer #1: **Yes:**    Nicolas Choquette-Levy

**********

[NOTE: If reviewer comments were submitted as an attachment file, they will be attached to this email and accessible via the submission site. Please log into your account, locate the manuscript record, and check for the action link "View Attachments". If this link does not appear, there are no attachment files.]

To ensure your figures meet our technical requirements, please review our figure guidelines: https://journals.plos.org/plosone/s/figures

You may also use PLOS’s free figure tool, NAAS, to help you prepare publication quality figures: https://journals.plos.org/plosone/s/figures#loc-tools-for-figure-preparation.

NAAS will assess whether your figures meet our technical requirements by comparing each figure against our figure specifications.

# Author comment: Author response to Decision Letter 1

**DOI:** 10.1371/journal.pone.0352242.r002

31 Mar 2026

See attached file

Attachment Submitted filename: ResponsetoReview.pdf

# Peer review: Decision Letter 1

**DOI:** 10.1371/journal.pone.0352242.r003

**Authors:** Asim Zia

19 May 2026

PONE-D-25-53063R1

A computational model of spatial politics: Hotelling-Downs model as statistical physics

PLOS One

Dear Dr. Ackland,

Thank you for submitting your manuscript to PLOS ONE. After careful consideration, we feel that it has merit but does not fully meet PLOS ONE’s publication criteria as it currently stands. Therefore, we invite you to submit a revised version of the manuscript that addresses the points raised during the review process.

Please submit your revised manuscript by Jul 03 2026 11:59PM. If you will need more time than this to complete your revisions, please reply to this message or contact the journal office at plosone@plos.org. When you're ready to submit your revision, log on to https://www.editorialmanager.com/pone/ and select the 'Submissions Needing Revision' folder to locate your manuscript file.

Please include the following items when submitting your revised manuscript:

- A letter that responds to each point raised by the academic editor and reviewer(s). You should upload this letter as a separate file labeled 'Response to Reviewers'.
- A marked-up copy of your manuscript that highlights changes made to the original version. You should upload this as a separate file labeled 'Revised Manuscript with Track Changes'.
- An unmarked version of your revised paper without tracked changes. You should upload this as a separate file labeled 'Manuscript'.

If you would like to make changes to your financial disclosure, please include your updated statement in your cover letter. Guidelines for resubmitting your figure files are available below the reviewer comments at the end of this letter.

If applicable, we recommend that you deposit your laboratory protocols in protocols.io to enhance the reproducibility of your results. Protocols.io assigns your protocol its own identifier (DOI) so that it can be cited independently in the future. For instructions see: https://journals.plos.org/plosone/s/submission-guidelines#loc-laboratory-protocols. Additionally, PLOS ONE offers an option for publishing peer-reviewed Lab Protocol articles, which describe protocols hosted on protocols.io. Read more information on sharing protocols at https://plos.org/protocols?utm_medium=editorial-email&utm_source=authorletters&utm_campaign=protocols.

As the corresponding author, your ORCID iD is verified in the submission system and will appear in the published article. PLOS supports the use of ORCID, and we encourage all coauthors to register for an ORCID iD and use it as well. Please encourage your coauthors to verify their ORCID iD within the submission system before final acceptance, as unverified ORCID iDs will not appear in the published article. *Only*    the individual author can complete the verification step; PLOS staff *cannot*    verify ORCID iDs on behalf of authors.

We look forward to receiving your revised manuscript.

Kind regards,

Asim Zia, Ph.D.

Academic Editor

PLOS One

Journal Requirements:

1. If the reviewer comments include a recommendation to cite specific previously published works, please review and evaluate these publications to determine whether they are relevant and should be cited. There is no requirement to cite these works unless the editor has indicated otherwise.

2. Please review your reference list to ensure that it is complete and correct. If you have cited papers that have been retracted, please include the rationale for doing so in the manuscript text, or remove these references and replace them with relevant current references. Any changes to the reference list should be mentioned in the rebuttal letter that accompanies your revised manuscript. If you need to cite a retracted article, indicate the article’s retracted status in the References list and also include a citation and full reference for the retraction notice.

Additional Editor Comments:

Many thanks for your thorough efforts to address comments from the first round of reviews. Reviewer 1 has recommended a few minor revisions that need to be addressed before formal acceptance.

[Note: HTML markup is below. Please do not edit.]

Reviewer's Responses to Questions

**Comments to the Author**

1. If the authors have adequately addressed your comments raised in a previous round of review and you feel that this manuscript is now acceptable for publication, you may indicate that here to bypass the “Comments to the Author” section, enter your conflict of interest statement in the “Confidential to Editor” section, and submit your "Accept" recommendation.

Reviewer #1: (No Response)

Reviewer #2: All comments have been addressed

**********

2. Is the manuscript technically sound, and do the data support the conclusions?

The manuscript must describe a technically sound piece of scientific research with data that supports the conclusions. Experiments must have been conducted rigorously, with appropriate controls, replication, and sample sizes. The conclusions must be drawn appropriately based on the data presented.

Reviewer #1: Yes

Reviewer #2: Yes

**********

3. Has the statistical analysis been performed appropriately and rigorously?

Reviewer #1: Yes

Reviewer #2: Yes

**********

4. Have the authors made all data underlying the findings in their manuscript fully available?

The PLOS Data policy requires authors to make all data underlying the findings described in their manuscript fully available without restriction, with rare exception (please refer to the Data Availability Statement in the manuscript PDF file). The data should be provided as part of the manuscript or its supporting information, or deposited to a public repository. For example, in addition to summary statistics, the data points behind means, medians and variance measures should be available. If there are restrictions on publicly sharing data—e.g. participant privacy or use of data from a third party—those must be specified.

Reviewer #1: Yes

Reviewer #2: Yes

**********

5. Is the manuscript presented in an intelligible fashion and written in standard English?

PLOS ONE does not copyedit accepted manuscripts, so the language in submitted articles must be clear, correct, and unambiguous. Any typographical or grammatical errors should be corrected at revision, so please note any specific errors here.

Reviewer #1: Yes

Reviewer #2: Yes

**********

6. Review Comments to the Author

Please use the space provided to explain your answers to the questions above. You may also include additional comments for the author, including concerns about dual publication, research ethics, or publication ethics. (Please upload your review as an attachment if it exceeds 20,000 characters)

Reviewer #1: Thank you to the authors for their revised manuscript and thoughtful responses to my comments. I think the authors have substantially strengthened the paper and clarified many of the questions I had in reading the original draft. In particular, their explanation of different types of equilibria is instructive, the contributions of the turnout and activists concepts in the model are much clearer, and the connections to observed stylized political facts are more convincing. The addition of different voter distributions – including multi-peaked distributions in two-dimensional space – provides an interesting new window into the model’s skill in describing different political phenomena. In general, I am enthusiastic about the publication of this manuscript in PLoS One.

I have a few remaining comments for the authors to consider. However, these are mostly minor and should not impede publication of the manuscript. I offer these mainly to help clarify some of the additional detail that has been added to this revised manuscript:

1. L33-35: This is a helpful description of different concepts of polarization in the Downs framework, and it would be useful to explicitly state which ones you are engaging with here (I think just the latter?).

2. L62-67: Similarly, this is a helpful description of different types of equilibria, and it would be good to state which one(s) you use in this analysis.

3. L138-141: I found this description to be especially helpful in understanding the connection between turnout and activism in the model. This level of detail rightly belongs in the “Model” section, but I wonder if this conceptual link could be made earlier in the Introduction. That is, could you more clearly motivate why it’s important to analyze turnout and activism jointly (e.g., pointing to the possibility that the effects of imperfect turnout on polarization are likely to depend on whether voters are uniformly weighted)? I think that might help readers more easily track the contributions of this multi-factor approach.

4. L58-173: This section is an interesting addition and makes sense to me statistically, but I was unclear about what “correlation” means in a political context here. Does this refer to correlation on a [-1,1] spectrum in voter perceptions on Issue X and Y? In other words, in a high-correlation condition, if voters tend to be more “left” on issue X, would they also be more “left” on issue Y? Perhaps an example here might help.

5. Table 1 caption: This last sentence (“The 2D data is … tending towards 0.765”) didn’t make sense to me. Why would a random placement necessarily tend to 0.765?

6. Fig. 2: This is an intriguing comparison of equilibria positions for different numbers of parties. I think the example trajectories on the left-hand panel are helpful, but (at risk of contradicting myself from the initial review) they might be overkill for the right-hand panel with considerably more parties. Additionally, it might be helpful to include arrows showing the direction of the movement (I assume generally from the outside towards the centre of the circle). Finally, the caption mentions a legend for final vote share, but I think this has been omitted, at least from the image files in this submission.

7. L 269-271: This is an interesting finding, and I wonder if there are also non-linearities in the turnout*activism interaction depending on the strength of the turnout factor. In other words, does activism change the critical value of \Tau for which polarization starts to decrease again? This might be grist for future work or reflections in the last part of the Discussion. But I thought it would be helpful to bring up here, as it might explain within-case study patterns whereby polarization increases even as voter turnout steadily decreases.

8. L 499-503: Slight quibble here. I understand the point that more parties may lead each individual party to be more extreme than a two-party system. But if parties on different ends of an issue are in a coalition government (arguably more likely in a system with several parties), I don't think this necessarily makes the government and government policy less representative of the overall median voter.

Reviewer #2: The authors have substantially improved the manuscript in response to the previous round of review. The revised version provides a clearer theoretical framing of the contribution, improves the explanation of the computational methodology, and offers a more balanced interpretation of the model’s implications for party competition and political polarisation. I particularly appreciate the stronger discussion of turnout effects, activist influence, multidimensional opinion space, and the distinction between voter and elite polarisation. The manuscript now does a better job situating the model within the broader literature on Downsian competition, spatial voting, and computational political science. The clarification of equilibrium concepts and the expanded discussion of limitations also strengthen the paper considerably.

The modelling framework is technically coherent, the simulation results are clearly presented, and the conclusions are generally well supported by the analyses. The availability of open-source code and computational materials is also an important strength that enhances transparency and reproducibility. Although the empirical component remains illustrative rather than causal, the manuscript now appropriately frames the comparative turnout–polarisation analysis as supportive evidence rather than definitive validation. The discussion is more cautious and proportionate to the scope of the model.

Overall, I believe the manuscript now makes a valuable interdisciplinary contribution linking statistical physics approaches with political competition theory and computational social science. I have no further substantive concerns and consider the manuscript suitable for publication.

**********

7. PLOS authors have the option to publish the peer review history of their article (what does this mean?). If published, this will include your full peer review and any attached files.

If you choose “no”, your identity will remain anonymous but your review may still be made public.

**Do you want your identity to be public for this peer review?**    For information about this choice, including consent withdrawal, please see our Privacy Policy.

Reviewer #1: **Yes:**    Nicolas Choquette-Levy

Reviewer #2: No

**********

[NOTE: If reviewer comments were submitted as an attachment file, they will be attached to this email and accessible via the submission site. Please log into your account, locate the manuscript record, and check for the action link "View Attachments". If this link does not appear, there are no attachment files.]

To ensure your figures meet our technical requirements, please review our figure guidelines: https://journals.plos.org/plosone/s/figures

You may also use PLOS’s free figure tool, NAAS, to help you prepare publication quality figures: https://journals.plos.org/plosone/s/figures#loc-tools-for-figure-preparation.

NAAS will assess whether your figures meet our technical requirements by comparing each figure against our figure specifications.

# Author comment: Author response to Decision Letter 2

**DOI:** 10.1371/journal.pone.0352242.r004

1 Jun 2026

1. L33-35: This is a helpful description of different concepts of polarization in the Downs framework, and it would be useful to explicitly state which ones you are engaging with here (I think just the latter?).

Agreed. It is the latter, although later we do also touch on differences between more or less steep single-peaked distributions.

2. L62-67: Similarly, this is a helpful description of different types of equilibria, and it would be good to state which one(s) you use in this analysis.

}

Agreed and added.

3. L138-141: I found this description to be especially helpful in understanding the connection between turnout and activism in the model. This level of detail rightly belongs in the “Model” section, but I wonder if this conceptual link could be made earlier in the Introduction. That is, could you more clearly motivate why it’s important to analyze turnout and activism jointly (e.g., pointing to the possibility that the effects of imperfect turnout on polarization are likely to depend on whether voters are uniformly weighted)? I think that might help readers more easily track the contributions of this multi-factor approach.

}

A sentence has been added to the introduction.

4. L58-173: This section is an interesting addition and makes sense to me statistically, but I was unclear about what “correlation” means in a political context here. Does this refer to correlation on a [-1,1] spectrum in voter perceptions on Issue X and Y? In other words, in a high-correlation condition, if voters tend to be more “left” on issue X, would they also be more “left” on issue Y? Perhaps an example here might help.

}

The referee understands it perfectly - statistical correlation in the 2D space. One might consider the Pearson correlation coefficient, but one can construct distribrution like Fig 3 with 3 or 4 peaks for which that formal measure is zero.

We give the following example:

By extending the opinion space to two dimensions, we can investigate the effects on voting if two different issues are important, e.g. Economic and Cultural on axes from left to right-wing and liberal/multicultural to traditional/nationalist.

Each party must take a position of both issues, for example a left wing, nationalist party.

The distribution of voters may then be {\it correlated}: e.g. there may be more left-wing nationalists than right-wing ones. We investigate such a correlated situation where

on both issues there is a consensus (most voters in the centre), but the two dimensional space may have two peaks and show that, with only two parties, the Hotelling-consensus emerges, but with more parties they move to the peaks of the voter distribution.

5. Table 1 caption: This last sentence (“The 2D data is … tending towards 0.765”) didn’t make sense to me. Why would a random placement necessarily tend to 0.765?}

Thank you for pointing that out - is indeed far from obvious. It involves the integral for the mean distance of a randomly chosen point from the centre of the square, which is $\frac{1}{3}(\sqrt{2}+\ln(1+\sqrt{2})=0.765$. We now added the equation.

6. Fig. 2: This is an intriguing comparison of equilibria positions for different numbers of parties. I think the example trajectories on the left-hand panel are helpful, but (at risk of contradicting myself from the initial review) they might be overkill for the right-hand panel with considerably more parties. Additionally, it might be helpful to include arrows showing the direction of the movement (I assume generally from the outside towards the centre of the circle). Finally, the caption mentions a legend for final vote share, but I think this has been omitted, at least from the image files in this submission.}

We thank the referee for spotting the error that the vote shares are not given: they vary significantly from run to run so specific numbers are not informative nor reproducible; for each case, they can be inferred from the shaded area. We deleted the clause.

We accept that the second figure with 20 parties is overkill for practical purposes, but we prefer to retain it to display a limiting case of the model. We explored using arrows, but we found that the "tadpole" plots with the head showing the final position are clearer, but to address the referees concern we have the explanatory text "Lines

show typical trajectories from random starting locations, circles show final positions,"

7. L 269-271: This is an interesting finding, and I wonder if there are also non-linearities in the turnout*activism interaction depending on the strength of the turnout factor. In other words, does activism change the critical value of \Tau for which polarization starts to decrease again? This might be grist for future work or reflections in the last part of the Discussion. But I thought it would be helpful to bring up here, as it might explain within-case study patterns whereby polarization increases even as voter turnout steadily decreases.}

This is an interesting question but it requires an additional parameter which essentially put the answer in. The model is flexible enough to allow for this, but we feel it should be grounded in some fieldwork so we leave it for future work.

\textit{8. L 499-503: Slight quibble here. I understand the point that more parties may lead each individual party to be more extreme than a two-party system. But if parties on different ends of an issue are in a coalition government (arguably more likely in a system with several parties), I don't think this necessarily makes the government and government policy less representative of the overall median voter.

Interesting point on which we now comment. This argument is based around the premise "parties on different ends of an issue are in a coalition government". This may apply to some issues, but in general coalitions are formed between parties with similar ideologies.

Attachment Submitted filename: OpinionSpaceResponse2.pdf

# Editor report: Decision Letter 2

**DOI:** 10.1371/journal.pone.0352242.r005

**Authors:** Asim Zia

8 Jun 2026

A computational model of spatial politics: Hotelling-Downs model as statistical physics

PONE-D-25-53063R2

Dear Dr. Ackland,

We’re pleased to inform you that your manuscript has been judged scientifically suitable for publication and will be formally accepted for publication once it meets all outstanding technical requirements.

Within one week, you’ll receive an e-mail detailing the required amendments. When these have been addressed, you’ll receive a formal acceptance letter and your manuscript will be scheduled for publication.

An invoice will be generated when your article is formally accepted. Please note, if your institution has a publishing partnership with PLOS and your article meets the relevant criteria, all or part of your publication costs will be covered. Please make sure your user information is up-to-date by logging into Editorial Manager at Editorial Manager® and clicking the ‘Update My Information' link at the top of the page. For questions related to billing, please contact billing support.

If your institution or institutions have a press office, please notify them about your upcoming paper to help maximize its impact. If they’ll be preparing press materials, please inform our press team as soon as possible -- no later than 48 hours after receiving the formal acceptance. Your manuscript will remain under strict press embargo until 2 pm Eastern Time on the date of publication. For more information, please contact onepress@plos.org.

Kind regards,

Asim Zia, Ph.D.

Academic Editor

PLOS One

Additional Editor Comments (optional):

All outstanding issues and comments have been adequately addressed in this version.

Reviewers' comments:

# Editor report: Acceptance letter

**DOI:** 10.1371/journal.pone.0352242.r006

**Authors:** Asim Zia

PONE-D-25-53063R2

PLOS One

Dear Dr. Ackland,

I'm pleased to inform you that your manuscript has been deemed suitable for publication in PLOS One. Congratulations! Your manuscript is now being handed over to our production team.

At this stage, our production department will prepare your paper for publication. This includes ensuring the following:

* All references, tables, and figures are properly cited

* All relevant supporting information is included in the manuscript submission,

* There are no issues that prevent the paper from being properly typeset

You will receive further instructions from the production team, including instructions on how to review your proof when it is ready. Please keep in mind that we are working through a large volume of accepted articles, so please give us a few days to review your paper and let you know the next and final steps.

Lastly, if your institution or institutions have a press office, please let them know about your upcoming paper now to help maximize its impact. If they'll be preparing press materials, please inform our press team within the next 48 hours. Your manuscript will remain under strict press embargo until 2 pm Eastern Time on the date of publication. For more information, please contact onepress@plos.org.

You will receive an invoice from PLOS for your publication fee after your manuscript has reached the completed accept phase. If you receive an email requesting payment before acceptance or for any other service, this may be a phishing scheme. Learn how to identify phishing emails and protect your accounts at https://explore.plos.org/phishing.

If we can help with anything else, please email us at customercare@plos.org.

Thank you for submitting your work to PLOS ONE and supporting open access.

Kind regards,

PLOS ONE Editorial Office Staff

on behalf of

Professor Asim Zia

Academic Editor

PLOS One
